# Evaluation of Novel Dillapiol Analogs as Insect Detoxification Enzyme Inhibitors and Insecticide Synergists †

**DOI:** 10.3390/insects17030351

**Published:** 2026-03-23

**Authors:** Suqi Liu, Ana Francis Carballo-Arce, Zhiling Wang, Tony Durst, Steven R. Sims, John T. Arnason, Ian M. Scott

**Affiliations:** 1 PEI Department of Agriculture, Charlottetown, PE C1A 7N8, Canada; sxliu@gov.pe.ca; 2 Department of Chemistry, Universidad Nacional Autonoma, Heredia 86-3000, Costa Rica; ana.carballo.arce@una.ac.cr; 3 Department of Forestry, Shanxi Agricultural University, Taigu, Jinzhong 030801, China; wzl1983@nwafu.edu.cn; 4 Chemistry Department and Biomolecular Sciences, University of Ottawa, D’Iorio Hall, 10 Marie Curie, Ottawa, ON K1N 6N5, Canada; tony.durst@uottawa.ca; 5 Blue Imago LLC, 1973 Rule Ave., Maryland Heights, MO 63043, USA; steve.sims@blueimago.com; 6 Biology Department, Faculty of Science, University of Ottawa, 30 Marie Curie, Station A, P.O. Box 450, Ottawa, ON K1N 6N5, Canada; john.arnason@uottawa.ca; 7 London Research and Development Centre, Agriculture and Agri-Food Canada, London, ON N5V 4T3, Canada

**Keywords:** dillapiol analogs, insecticide synergists, P450 detoxification enzymes, glutathione S-transferases

## Abstract

Dillapiol is a naturally occurring methylenedioxyphenyl compound with insecticide-synergizing activity comparable to piperonyl butoxide (PBO). This study identified structurally related molecules with practical potential for managing insecticide-resistant insects. Six new dillapiol analogs, containing ester- or ether-linked side chains, were synthesized and evaluated as pyrethrum synergists against the Colorado potato beetle (CPB) *Leptinotarsa decemlineata* (Say) (Coleoptera: Chrysomelidae). Their activity was assessed through bioassays and by quantifying inhibition of Phase I and II detoxification enzymes in vitro and in vivo. All six compounds displayed higher synergistic activity by ingestion than by topical exposure, and each structural class included at least one compound with a synergism ratio greater than 20. In the resistant CPB strain (RS-CPB), two ester compounds inhibited P450 monooxygenase activity in vitro as effectively as PBO, while dillapiol and one ether analog reduced P450 activity in vivo. Notably, all six analogs reduced glutathione S-transferase (GST) activity; the most active was an ether analog with an in vitro IC_50_ of 0.23 (±0.04) mM. Dillapiol also significantly reduced GST activity in vivo. These analogs demonstrated PBO-equivalent P450 inhibition combined with unique GST inhibition and show promise as alternative synergists for managing insecticide-resistant insects.

## Simple Summary

This study was designed to address the critical challenge of widespread insecticidal resistance in agricultural pests by exploring novel botanical synergists as alternatives to piperonyl butoxide (PBO), a primary insecticide synergist in today’s market but facing growing safety concerns due to adverse mammalian effects. Our study focused on dillapiol, a natural botanical compound with synergistic efficacy comparable to PBO. We synthesized six novel dillapiol analogs and evaluated their ability to enhance the potency of pyrethrum against the Colorado potato beetle (CPB). Several analogs significantly boosted pyrethrum’s insecticidal activity, especially when the compounds were consumed by insects rather than topically applied. Mechanistic investigations confirmed that, like PBO, several new compounds effectively inhibited key Phase I detoxification enzymes. These enzymes perform the primary transformation of molecules through oxidation–reduction to create water-soluble compounds. Notably, several new analogs demonstrated higher inhibitory activity of the analogs compared to PBO in the enzyme inhibition assays. Some even demonstrated a unique capacity to reduce Phase II detoxification enzyme activity, which facilitates the conjugation of molecules into water-soluble forms, a process typically unaffected by PBO in in vivo assays. One compound, in particular, exhibited a significant reduction in enzymatic activity across both phases. Our data suggest that these new dillapiol-based compounds represent a promising new class of synergists. Their enhanced efficacy and novel modes of action could enhance the management of resistant pest populations, improve integrated pest management (IPM) strategies and contribute to more sustainable agricultural practices.

## 1. Introduction

Synergists are additives used to increase the efficacy and reduce the application rates of insecticides. They have been in commercial use for more than 50 years. Among insecticide synergists, compounds containing the methylenedioxyphenyl (MDP) moiety are most widely utilized due to their potent inhibition of insect P450 detoxification enzymes. Today, the most common insecticide synergist is the MDP compound, piperonyl butoxide (PBO), which is used in most pyrethrin formulations registered for agricultural, commercial, public health, and household uses in North America. In 2023, the global PBO market was valued at approximately $349.2 million, and it is projected to reach $501.3 million by 2030 [1]. Within this market, the home pest care segment is the largest, accounting for 40% of production, followed by agriculture at 35% [1]. The Asia Pacific PBO market leads, accounting for about 44% of the global share [1]. However, exposure to PBO may induce short- or long-term adverse effects in mammals [2]. These include impairment of liver function [3], murine liver hypertrophy [4], and negative effects on the nervous system [5,6]. PBO may also be a carcinogen [7]. Due to these toxicology issues, PBO replacements are needed.

Dillapiol is a naturally occurring MDP compound existing in a variety of tropical plants, including wild pepper species such as *Piper aduncum* L. It has proven to be an effective insecticide synergist [8,9,10,11]. A previous study with more than 20 dillapiol analogs developed a model based on lipophilicity and molecular refractivity that predicted synergistic mosquito larvicidal activity in combination with α-terthienyl [12].

More than 55 dillapiol analogs were prepared by semisynthesis using dillapiol and sesamol as the starting materials [13] and evaluated using an in vitro human CYP3A4 P450 enzyme assay [14]. Based on mammalian P450 inhibition, three ester compounds with activity comparable to dillapiol were selected for insect evaluation. Considering that insect esterase enzymes might affect the performance and longevity of the ester compounds in vivo [15], as well as being a mechanism of insecticide resistance in some insect pests [16,17,18,19], three ether analogs resistant to hydrolysis by esterases were also studied. All compounds were evaluated as potential pyrethrum synergists and tested on a challenging insect model, a multiple insecticide-resistant population of the Colorado potato beetle (CPB), *Leptinotarsa decemlineata* (Say) (Coleoptera: Chrysomelidae).

The CPB is the most important pest of potatoes worldwide due to its ability to rapidly develop resistance to every new class of insecticide that has been used to control it. The mechanisms involved in the insecticide resistance of the CPB include enhanced activity of monooxygenases [20], esterases [21], or carboxylesterases [22]. The objectives of the present study were (1) to determine the potential of six selected dillapiol analogs as pyrethrum synergists, and (2) to study their inhibitory effects on major detoxification enzymes present in an insecticide-resistant CPB strain.

## 2. Materials and Methods

### 2.1. Insects

Insecticide-resistant Colorado potato beetles (RS-CPBs) were obtained from a colony originating from Long Island, New York, a population previously characterized by Olson et al. [23] to exhibit high imidacloprid tolerance associated with enhanced excretion. The colony was maintained at the Department of Entomology, Michigan State University, East Lansing, MI, USA, prior to transfer to the London Research and Development Centre (LRDC), Agriculture and Agri-Food Canada (AAFC), London, ON, Canada. Upon arrival, the RS-CPB were reared for >20 generations without exposure to insecticides before use in the bioassays and biochemical assays. Insecticide-susceptible CPBs (SS-CPBs) were obtained from a colony reared for >150 generations without insecticide exposure at the AAFC London laboratory. Both CPB strains were reared on greenhouse-grown potato *Solanum tuberosum* L. (Var. Kennebec) foliage and maintained in an insectary at 27 ± 1 °C, 65 ± 5% RH, and a photoperiod of 16:8 h L:D.

### 2.2. Insecticide and Synergist Preparation

**Figure 1:**
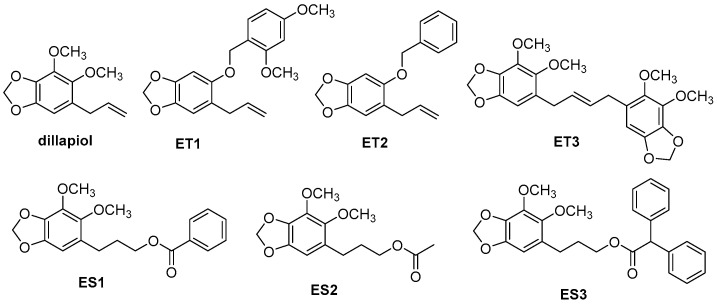
Chemical structure of dillapiol and its six analogs. ES1–ES3 are ester compounds. ET1–ET3 are ether compounds.

The sources of pyrethrum and dillapiol were previously described [11]. Briefly, pyrethrum extract was obtained from Whitmire Micro-Gen (BASF, St. Louis, MO, USA) and purified by supercritical fluid extraction with CO_2_ at Loyalist College (Belleville, ON, Canada). Dillapiol was extracted by steam distillation from the fruit of *P. aduncum*, originally collected in the Sarapiqui region of Costa Rica, and purified using column chromatography. Dillapiol analogs were designed and synthesized from dillapiol as the starting material (Figure 1). First, dillapiol was converted to its primary alcohol via hydroboration. This alcohol was then esterified with various aliphatic and aromatic acids to produce a library of 17 ester analogs. To explore the role of the dillapiol methoxy group, additional analogs were prepared from sesamol. Sesamol was allylated and then underwent a Claisen rearrangement to form a key intermediate. This intermediate was used to generate 14 ethers and 10 esters with diverse structural features. All final compounds were purified, and their structures were confirmed by NMR spectroscopy and high-resolution mass spectrometry [13]. Based on previous CYP3A4 inhibition results, six analogs were selected for further testing in the present study.

For insect toxicity bioassays, all chemicals were dissolved in acetone, and the different solutions were prepared by combining pyrethrum and dillapiol or the analogs at a 1:5 ratio (wt/wt) [11]. This ratio enables direct comparison with our previous dillapiole studies. To test enzyme inhibition activity, compounds were dissolved either in acetone for in vivo assays or methanol for in vitro assays (solvents purchased from Sigma-Aldrich, Oakville, ON, Canada) to obtain a range of concentrations.

### 2.3. Insect Bioassays

Toxicity bioassays were conducted using previously described methods [11] with slight modifications. Two insecticide exposure methods, topical and oral, were used to test the synergistic potential of dillapiol analogs with a pyrethrum discriminating concentration and a range of pyrethrum concentrations against the RS-CPB.

The topical application bioassay was conducted by cutting 4 cm diameter leaf disks from potato leaves collected from greenhouse-grown plants. Five 2nd instar RS-CPB larvae were transferred to each leaf disk, and a 1 µL dose of the test solutions was applied to the dorsal thoracic region of the larvae using a 5 μL micro-applicator (Hamilton Robotics, Reno, NV, USA). Due to the limited amount of each analog available for testing (at the mg level), only three replicates were performed for each bioassay. A total of 210 larvae were used in the topical application bioassay. This comprised 14 treatments (analog–pyrethrum combinations) with 15 larvae per treatment (3 replicates × 5 larvae per replicate). RS-CPB larvae treated with acetone only served as controls. The treatment as well as control larvae were held under the same conditions in a holding room at 27 ± 1 °C, 65 ± 5% RH and a photo period of 16:8 h L:D. Mortality was recorded at 24 and 48 h by gently touching the larvae with a probe to assess response and mobility. Moribund larvae, which appeared to be dead but made occasional leg movements, were scored as dead.

For the ingestion method, each leaf disk was dipped for 3 s into the same solutions prepared for the topical method and dried in a fume hood for 20 min. Once dry, each disk was transferred to a Petri dish with a filter paper disk (Whatman #1) underneath. Control treatments consisted of leaf disks dipped in acetone alone. Five larvae were then placed on each leaf disk, and the Petri dishes were moved to a holding room for the duration of the study. A total of 360 2nd instar RS-CPB larvae were tested across 24 treatments. Each treatment was performed in triplicate with five larvae for each replicate (24 treatments × 3 replicates × 5 larvae per replicate). Mortality was assessed at 24 h, as described previously. Moribund larvae were scored as dead.

Discriminating concentration (DC) bioassays were used to compare the synergistic potential of dillapiol and its analogs with two diagnostic concentrations of pyrethrum. Three different pyrethrum concentrations at 10, 20, or 30 ppm were applied to test the ingestion toxicity of the pyrethrum/synergist combination, while pyrethrum concentrations of 50 and 100 ppm were chosen for topical treatments, to provide less than 100% mortality among all treatments. All pyrethrum concentrations were tested alone as controls for the synergist treatments.

The median lethal concentration (LC_50_) of pyrethrum alone or pyrethrum plus each of the different synergists using a full concentration bioassay was determined using a serial set of six concentrations of the different solutions made by the two-fold serial dilution method. Two separate series of bioassays were conducted to determine the LC_50_ values for each pyrethrum/synergist combination. A total of 30 larvae for each concentration (2 bioassays × 3 replicates × 5 larvae per replicate) were used, with a total of 180 larvae for each tested solution. The synergism ratio (SR) was estimated by dividing the LC_50_ of pyrethrum alone by the LC_50_ of pyrethrum plus synergists at the 1:5 ratio. The highest concentration of each synergist applied in the combination was also tested singly to determine the inherent toxicity to the RS-CPB in both topical and ingestion treatments.

### 2.4. Enzyme Assays

Fourth instar larvae of the RS-CPBs or SS-CPBs were used as a source of enzymes for in vitro and in vivo biochemical assays to test the enzyme inhibitory activity of each compound. The in vitro assay measured enzyme inhibitory activity in a reaction system containing pooled RS-CPB larval enzyme homogenate and the synergist. As for the in vivo enzyme assay, Samadieh et al. [24] demonstrated that the most statistically significant inhibition occurred 24 h after pre-treatment with the tested synergist. Therefore, SS- or RS-CPB larvae were topically pre-treated with the synergist 24 h prior to enzyme homogenate preparation, and then the enzymes were pooled, and the activity was measured. The enzymes for the different treatments were normalized to the protein concentration as determined by a BCA protein assay (Thermo Scientific, Mississauga, ON, Canada).

#### 2.4.1. Cytochrome P450 Assay (E.C. Number: 1.14.-.-)

Preparation of the CPB monooxygenase enzyme was done using the method of Scott et al. [25] with slight modifications. Specifically, 20 RS-CPB 4th instar larval midguts and fat bodies were dissected, and the gut food bolus was removed. The collected tissues were transferred to 1 mL of freshly prepared homogenization medium (HM). The HM was composed of 50 mL glycerol (40%), 1 mL ethylene diamine tetra acetic acid (EDTA) (100 mM), 1 mL dithiothreitol (DTT) (10 mM), 1 mL phenylmethyl sulfonylfluoride (PMSF) (100 mM), 1 mL phenylthiourea (PTU) (100 mM), and 71 mL 0.1 M sodium phosphate (NaP) buffer with pH 7.5 for a total volume of 100 mL. The CPB tissue and HM solution were homogenized at 1000 rpm for 1 min and centrifuged (Avanti J-26XP Beckman, Mississauga, ON, Canada) at 10,000× *g* for 20 min at 4 °C. The supernatant was collected and centrifuged (Optima XE, Beckman, Mississauga, ON, Canada) at 100,000× *g* for 1 h at 4 °C. The pellet was re-dissolved in a 2 mL re-suspension medium (RM). RM was prepared with 50 mL glycerol (40%), 1 mL EDTA (100 mM), 1 mL DTT (10 mM), 1 mL PMSF (100 mM), and 47 mL NaP buffer (0.1 M, pH 7.5) for a total volume of 50 mL. The microsome homogenate solution was stored at −80 °C until use.

The microsomal P450 activity was determined using the 7-methoxyresorufin O-demethylation (MROD) assay, where methoxyresorufin is converted to resorufin by monooxygenases in the presence of NADPH and oxygen [25]. The resorufin produced was measured fluorometrically in a 96-well microplate spectrofluorometer (Applied Biosystems, Foster City, CA, USA) at 530/590 nm excitation/emission. Each microplate well contained 202 µL of reaction mixture, which included 10 µL enzyme (1.5 mg/mL protein content), 10 µL synergist solution (5 µg/mL in methanol), 0.4 µL methoxyresorufin (1 mM), 2 µL NADPH (0.01 M) and 179.6 µL potassium phosphate buffer (0.1 M, pH 7.8). All solutions except NADPH were added to each well, and the microplate was incubated for 3 min at 30 °C. The reaction was initiated by the addition of NADPH. The percent inhibition by dillapiol or analog was determined relative to resorufin produced in the presence of the methanol control. PBO at the same concentration was used as the positive control.

#### 2.4.2. GST Enzyme Assay (EC Number: 2.5.1.18)

The enzyme preparation method was the same as that used for the monooxygenase enzyme homogenate preparation, except that HM and RM were replaced by a sodium phosphate (Sigma-Aldrich) buffer (pH 7.8, 0.1 M sodium phosphate), and, following centrifugation at 100,000× *g* for 1 h, the supernatant rather than the pellet was collected and stored at −80 °C as the enzyme stock.

The effect on GST activity was determined by measuring the change in absorbance at 340 nm produced by the conjugation of the glutathione thiol group of GSH (Sigma-Aldrich) to the 1-chloro-2, 4-dinitrobenzene (CDNB) (Sigma-Aldrich) substrate [26] using a Cytofluor 4000 Fluorescence Measurement System 96-well microplate reader (Applied Biosystems, Foster City, CA, USA). Briefly, 128 µL of solution A (50 µL of 0.1 M sodium phosphate buffer, pH 6.5, 70 µL of 1.5 mg/mL enzyme) and 8 µL synergist solution (dissolved in acetone at 1 mg/mL) were added to each well and incubated for 10 min at 30 °C. The absorbance was measured at 340 nm for 7 min as soon as an additional 172 µL of solution B (163 µL of 0.1 M sodium phosphate buffer, 3 µL GSH and 6 µL CDNB) was added to each well. Diethyl maleate (DEM) (Sigma-Aldrich, St. Louis, MO, USA), an inhibitor of GST [27], was included in the in vitro experimental design as a positive control to confirm that the experimental conditions were appropriate for detecting GST activity. The IC_50_ of DEM was also determined with the same volume in the reaction system, and boiled enzyme served as the negative control.

### 2.5. Statistical Analyses

The sample size, alpha level (0.05) and effective size f (0.25) were used to perform a statistical power analysis for the experiment based on three replicates using G*Power software (G*Power 3.1.9.2, Institute for Experimental Psychology, Dusseldorf, Germany). In all bioassay experiments, results were accepted only when mortality in the control group was less than 6.7% (i.e., no more than 1 dead larva out of 15 across three replicates). If mortality occurred in the control group, the observed mortality in the treatment groups was adjusted using Abbott’s formula to account for natural mortality. Probit analysis was performed using SAS software version 2.03 (SAS Institute, Cary, NC, USA, 2001) to generate regression equations, LC_50_ estimates, and 95% confidence limits (CL) for the SS- and RS-CPB strains under each pyrethrum/synergist combination. Due to limited quantities of the analog compounds, full dose–response curves for synergistic ratio calculations could not be generated. Therefore, to compare the fixed-dose activity of the analogs against the reference synergist (dillapiol and PBO), the general Linear Model, one-way ANOVA, and Bonferroni post hoc tests were conducted using Graphpad Prism 5.01 software (GraphPad Software, Inc., San Diego, CA, USA). Prior to ANOVA, synergistic activity data and enzyme inhibition activity data were log-transformed and arcsin-transformed, respectively, to meet the assumptions of normality. For IC_50_ and LC_50_, any two values compared were considered significantly different if their respective 95% CL did not overlap [28].

## 3. Results

### 3.1. Synergistic Effect of Dillapiol Analogs

The DC bioassays were conducted with two or three different concentrations for topical or ingestion treatments, respectively, to ensure the larval mortality ranged between 0 and 100% for all test analogs. The statistical power of the experiments was calculated for the synergistic activity bioassays based on three replicates per treatment, a sample size of 210 (topical treatment) or 360 (ingestion treatment), an alpha level of 0.05 and an effective size f of 0.25. We were able to detect differences in toxicity of different pyrethrum/analog combinations with an actual power of 0.8 and 0.81, respectively. The limited amount of each analog available for testing provided acceptable replication, based on the statistical power calculated for the number of treatments and replicates used in the synergist experiments.

**Figure 2:**
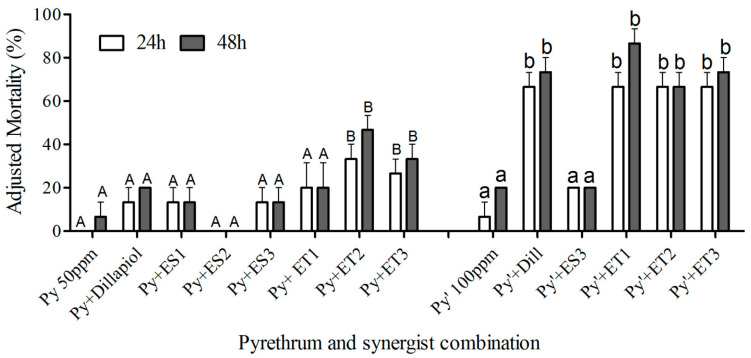
Percent mortality (±S.E.) of RS-CPB 2nd instar larvae 24 and 48 h after topical application of pyrethrum singly at 50 and 100 ppm or combined with synergists at 1:5 ratio. Bars with a different upper-case or lower-case letter from that of pyrethrum singly at 50 or 100 ppm, respectively, and other pyrethrum–synergist combinations indicate which combinations have significantly greater (*p* < 0.05) mortality.

Statistical analysis with integrated data determined that the two ether dillapiol analogs, ET2 and ET3, combined with 50 ppm pyrethrum and applied topically to RS-CPB larvae, produced significantly greater mortality at 24 and 48 h than pyrethrum alone (d.f. = 13,56; F = 37.08; *p* < 0.05) (Figure 2). Similarly, when the pyrethrum concentration was increased to 100 ppm, dillapiol and all three ether analogs produced significantly higher mortality than pyrethrum alone (Figure 2). All other dillapiol analogs tested by the topical method increased mortality, but not significantly more than pyrethrum alone (*p* > 0.05).

**Figure 3:**
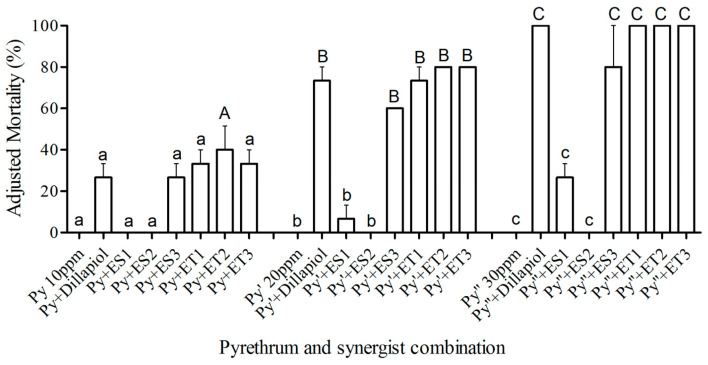
Percent mortality (±S.E.) of RS-CPB 2nd instar larvae 24 h after ingestion of pyrethrum singly at 10, 20, and 30 ppm or combined with synergists at 1:5 ratio. Bars with an upper-case letter (A, B or C) indicate synergist combinations that have significantly greater (*p* < 0.05) mortality than for pyrethrum singly at 10, 20, or 30 ppm, respectively, and other pyrethrum–synergist combinations (identified with the same lower-case letter).

When RS-CPB larvae were fed potato leaf disks pre-treated with 20 or 30 ppm pyrethrum plus synergists, there was significantly higher mortality with dillapiol, ES3 ester analog, and all three ether analogs, ET1, ET2 and ET3, compared to pyrethrum alone, while only ET2 produced significantly higher mortality when combined with pyrethrum at 10 ppm (d.f. = 23,48; F = 40.49; *p* < 0.05) (Figure 3). Ingestion of the esters, ES1 and ES2, did not significantly increase mortality (*p* > 0.05). These bioassays indicated that the three ether compounds possess greater potential as pyrethrum synergists than the ester compounds.

When dillapiol and the ET2 and ES3 analogs were combined with six pyrethrum concentrations at a 5:1 ratio, the resulting LC_50_ was lower, in all cases, than pyrethrum alone, and the synergism ratio (SR) ranged from 4 to >20 (Table 1). All compounds showed a higher SR by ingestion than by topical exposure.

### 3.2. Inhibitory Effect of Dillapiol Analogs on Phase I and II Metabolic Enzymes

The mode of action of dillapiol and its six analogs was evaluated by determining the in vivo and in vitro inhibitory activity to P450 monooxygenases and GSTs in RS-CPBs.

**Figure 4:**
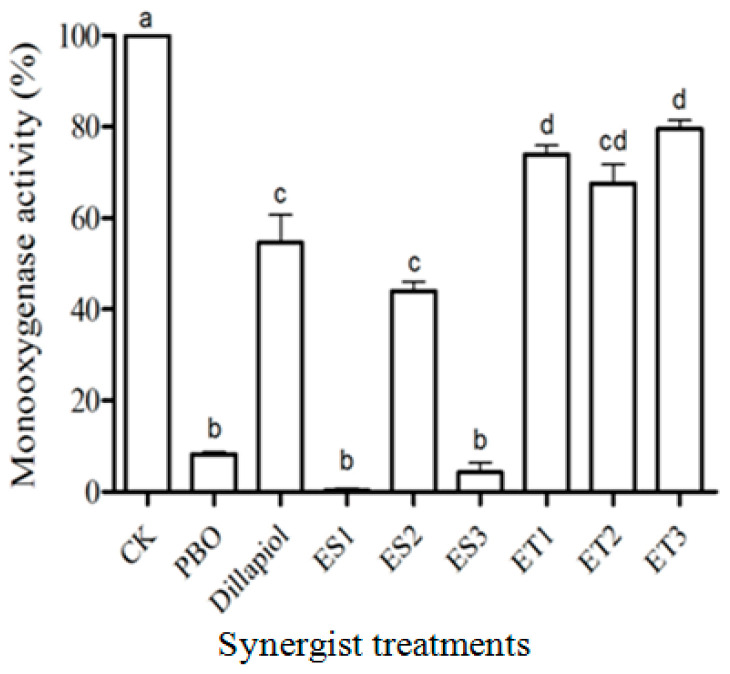
In vitro MROD monooxygenase residual activity (mean ± S.E.) in RS-CPB microsomes treated with solvent only (CK) or eight synergists (5 μg/mL). Different lowercase letters above the bars denote significant differences (*p* < 0.05) in monooxygenase activity among treatments.

**Figure 5:**
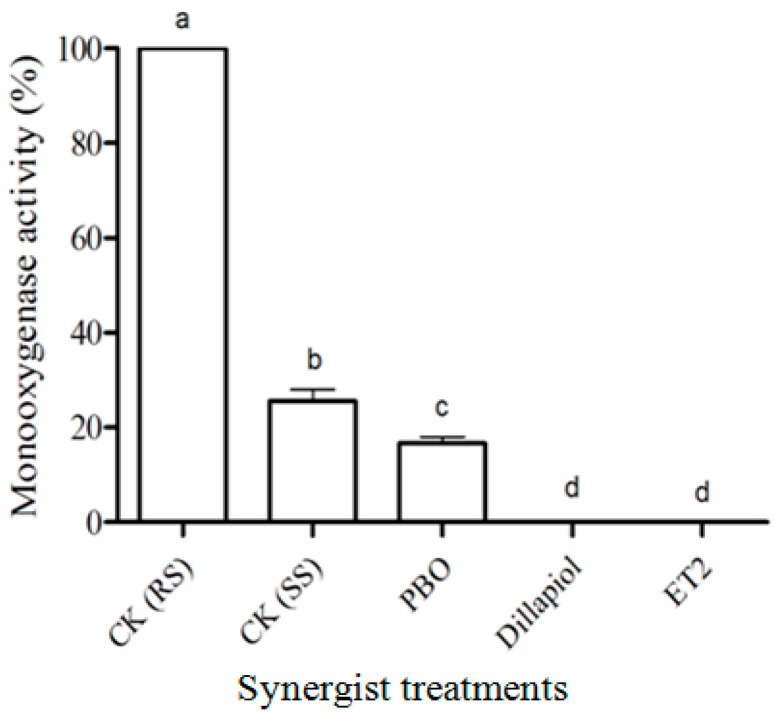
In vivo MROD monooxygenase residual activity (mean ± S.E.) in RS-CPB pre-treated with solvent only (CK RS) or three synergists (10 mg/mL). MROD activity in SS-CPB (CK SS) acted as a negative control. Different lowercase letters above the bars indicated significant differences (*p* < 0.05) in monooxygenase activity among treatments.

Ester compounds, ES1 and ES3, inhibited in vitro RS-CPB MROD monooxygenase activity as effectively as PBO at 5 µg/mL, while dillapiol, ES2, ET1, ET2 and ET3 were less active (d.f. = 8,18; F = 168.7; *p* < 0.05) (Figure 4). When RS-CPB were topically pre-treated with PBO, dillapiol and ET2, the in vivo monooxygenase activity decreased significantly (d.f. = 4,10; F = 1052; *p* < 0.05) (Figure 5). The cytochrome P450 activity of RS-CPB larvae was more than four times greater than that of the SS-CPB, but in vivo treatment with dillapiol and ET2 effectively inhibited this increased activity (Figure 5).

**Figure 6:**
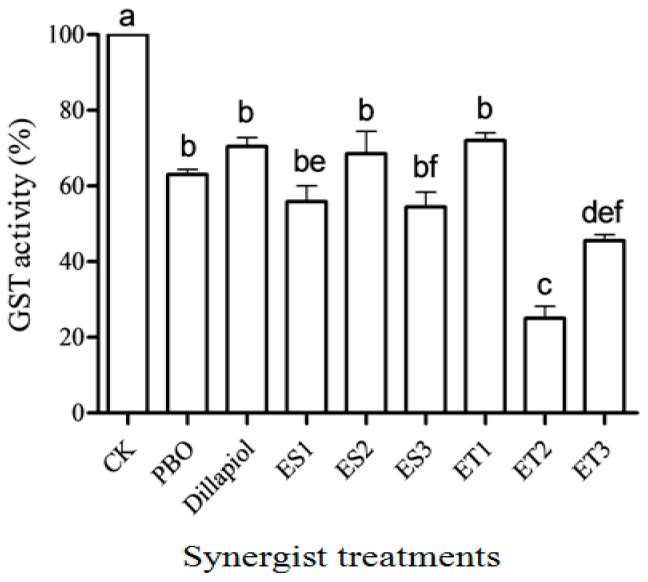
In vitro GST residual activity (mean ± S.E.) in RS-CPB enzyme homogenate treated with solvent only (CK) or eight synergists (1 mg/mL). Different lowercase letters above the bars indicated significant differences (*p* < 0.05) in GST activity among treatments.

In the in vitro GST activity assay, the mean DEM IC_50_ for three GST assays was determined as 42 (±3.28) mM. All dillapiol analogs at 1 mg/mL significantly reduced in vitro GST activity (d.f. = 8,18; F = 41.75; *p* < 0.05) (Figure 6). ET2, the most effective pyrethrum synergist, also displayed the most reduction in GST activity compared to all tested compounds, with an IC_50_ of ET2 at 0.23 (±0.04) mM.

**Figure 7:**
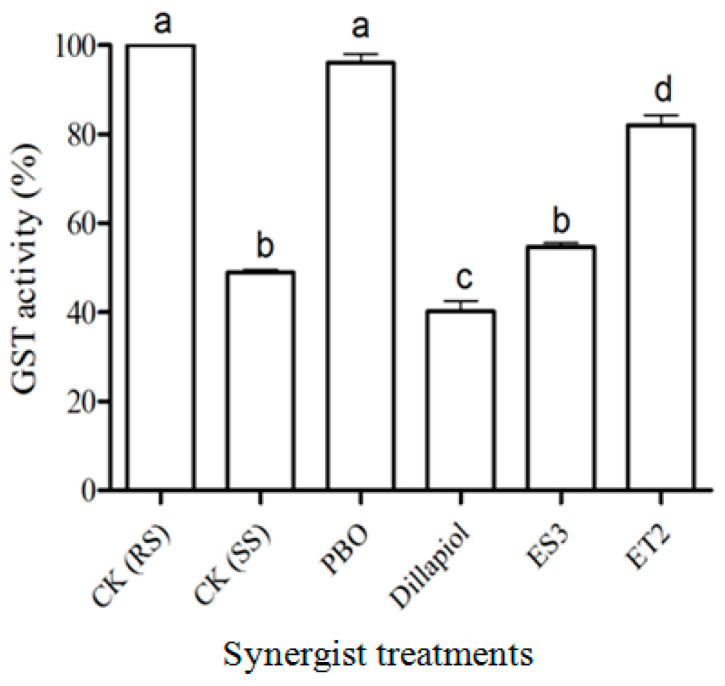
In vivo GST residual activity (mean ± S.E.) in RS-CPB pre-treated with solvent only (CK RS) or four synergists (10 mg/mL). GST enzyme activity of SS-CPB (CK SS) acted as negative control. Different lowercase letters above the bars indicated significant differences (*p* < 0.05) in GST activity among treatments.

Ether compounds ET2 and ET3 also reduced GST activity more than PBO in both in vitro and in vivo assays (*p* < 0.05). Insects pre-treated with different analogs had significantly reduced in vivo GST activity (d.f. = 5,12; F = 254.7; *p* < 0.05) except for those pre-treated with PBO (Figure 7). The residual GST activity in dillapiol-treated RS-CPB insects was significantly lower than for untreated SS-CPBs (*p* < 0.05).

## 4. Discussion

Plants represent a valuable source of biologically active natural products, and lead molecules selected for pharmaceutical or agricultural applications are often structurally optimized to enhance efficacy or reduce negative impacts. Dillapiol, a natural synergist, presents a potentially lower risk profile compared to the commercial standard, piperonyl butoxide (PBO), as it currently lacks reports of analogous adverse health effects. Nevertheless, a comprehensive toxicological evaluation of dillapiol and its analogs remains essential prior to product development.

In a previous study utilizing the same resistant CPB strain described here, dillapiol was demonstrated to increase pyrethrum insecticidal efficacy by 9.1-fold [11]. This pronounced synergism is likely attributable to the inhibition of enhanced detoxification enzymes in the resistant strain, a hypothesis supported by the substantially lower (2.2-fold) synergism observed in the susceptible CPB strain [11]. These findings established dillapiol as a viable benchmark for the development of novel synergists.

In the present study, we sought to identify dillapiol analogs with improved synergistic potential. Initial screening of dillapiol ester analogs via CYP3A4 isozyme inhibition had previously identified two promising candidates, ES1 and ES3 [14]. Here, we demonstrate that these compounds exhibit significantly greater inhibitory activity than dillapiol in an insect in vitro monooxygenase assay (Figure 4), confirming the value of this structural optimization approach. However, only ES3 showed comparable activity to dillapiol in both mortality assays (Figure 2 and Figure 3). The relatively strong activity of ES3 compared to ES1 and ES2 is likely due to its decreased susceptibility to the esterase enzymes resulting from increased steric hindrance at the carbonyl carbon. Since esters are susceptible to esterase-induced hydrolysis, comparable ether compounds were designed, and two of these, ET2 and ET3, were more active than dillapiol in the topical mortality assay. In any case, there is still an increased synergism factor for both the ester ES3 (15.8) and the ether ET2 (27) relative to dillapiol (5.8) (Table 1). These compounds lowered the pyrethrum concentration required for the RS-CPB control to a level effective against the SS-CPB (LC_50_ = 11 ppm) [11]. Dillapiol and the selected analogs, therefore, restored the pyrethrum susceptibility of the RS-CPB strain.

The toxicity differences observed for the dillapiol analogs in topical and ingestion assays (Figure 2 and Figure 3), and the lower LC_50_s by ingestion than topical application (Table 1), are probably due to differences in toxicokinetics. Although strong feeding deterrent effects occurred when pyrethroids were applied at 50 ppm in no-choice feeding tests against fall armyworm (*Spodoptera frugiperda*) [29], the feeding deterrence was decreased at a lower insecticide concentration (10 ppm), similar to the levels tested in the present study. In the topical treatment, insect cuticle, regarded as a barrier to compound uptake [30], is likely contributing to the higher LC_50_s compared to the LC_50_s determined through ingestion in the present study. Since the mesothoracic spiracle plays an important role in moderating the pyrethroid quick knockdown effect, it suggests that an improvement of spray application technology that allows insecticide particles to adhere efficiently to the insect and then enter rapidly into the body [31], and could potentially enhance the efficacy of pyrethrum/synergist solution by topical treatment. PBO possesses more than 30-fold synergism for pyrethrum against resistant CPBs [11], which is greater than any analog tested in the present study. PBO shows greater synergism in the insecticide bioassay through increased mortality than the dillapiol analogs, but less monooxygenase inhibition activity. The explanation for the difference could be the surfactant properties of PBO, which can enhance cuticular penetration of the pyrethrum [32]. Although the dillapiol analogs are not significantly better than PBO, they merit consideration as synergist replacements if PBO registration is cancelled for toxicological or other reasons [3,4,5,6,7].

Since MDP compounds are known to be effective P450 inhibitors, it was not surprising that all the tested compounds showed significant monooxygenase inhibition activity at a relatively low concentration (5 µg/mL). The ester analogs, ES1 and ES3, possessed P450 inhibition activity similar to PBO during in vitro testing (Figure 4), but the ether compounds were less effective. Since ET2, the most effective synergist in the toxicity bioassay, also produced significant monooxygenase inhibitory activity in both the in vitro (Figure 4) and in vivo (Figure 5) assays, we suggest that P450s play an important role in the CPB insecticide resistance. The lower SR value of ES1 and ES3 in the toxicity bioassay might be due to lower cuticle penetration ability or the hydrolysis of ester compounds in the insects.

In addition to elevated monooxygenase activity, GST activity in the RS-CPB was two times higher than in the SS-CPB (Figure 7), which is consistent with other RS-CPB research [27,33] and studies on other insects [34,35]. These results indicated that GST plays a role in the mechanism of CPB insecticide resistance. In the present study, PBO did not reduce GST activity in vivo, but dillapiol and its analogs significantly reduced GST activity in vitro and in vivo (Figure 6 and Figure 7) of the RS-CPB to the level measured in the SS-CPB. The most promising analogs were the two ether compounds, ET2 and ET3, which had the highest synergistic activity in the toxicity bioassays as well as the most reduction in in vitro and in vivo GST activity (Figure 6). This is the first report of reduced GST activity by dillapiol or related compounds containing the MDP group (see Table 2 for a summary of all analogs that caused synergism or no effect on pyrethrin toxicity in the bioassays, as well as synergism/inhibition status in the in vitro and in vivo enzyme assays). The implications of this finding related to the potential use of these compounds as pyrethrum synergists are important since GSTs may play a role in pyrethrin metabolism in different organisms, such as pyrethroid-resistant strains of the European corn borer (*Ostrinia nubilalis*) [36], silkworm (*Bombyx mori*) [37], rainbow trout (*Oncorhynchus mykiss*) [38], and red spider mite (*Oligonychus coffeae*) [39].

## 5. Conclusions

Several dillapiol analogs effectively synergized the biological activity of pyrethrum against insecticide-resistant CPBs under a range of test concentrations. The synergistic activity of these compounds may be conferred by their additive inhibition function with two different detoxification enzyme groups, microsomal P450s and GSTs. Dillapiol analogs show promise as new insecticide synergists that could replace PBO with the additional feature of reducing GST activity. This could delay the evolution of resistance in pest insect populations.

## Figures and Tables

**Table 1:** LC_50_ values and 95% confidence intervals (CI) of pyrethrum (Py) alone or in combination with dillapiol or two analogs, 24 h post treatment. The synergism ratio (SR) was determined relative to Py for 2nd instar RS-CPB larvae by topical or ingestion treatments.

Insecticide with or Without Synergists	Topical Treatment		Ingestion Treatment	
	LC_50_ (95% C.I.) (ppm)	Synergism Ratio (SR)	LC_50_ (95% C.I.) (ppm)	Synergism Ratio (SR)
Py	635 (584.8, 691.8)	N/A	225.5 (182.8, 282.5)	N/A
Py + Dillapiol	97.5 (76.5, 121.9)	6.5	14.3 (3, 21.8)	15.8
Py + ES3	153.5 (132.7, 185.8)	4.1	11.3 (3, 22.7)	20
Py + ET2	93.4 (81.6, 107.4)	6.8	8.3 (6.2, 14.1)	27

**Table 2:** Synergistic activity and enzyme inhibition effects of dillapiol analogs against resistant Colorado potato beetle (RS-CPB).

Compound	Structure Type	Topical Synergism ^a^	Ingestion Synergism ^a^	SR Value (Ingestion) ^b^	In Vitro P450 Inhibition ^c^	In Vivo P450 Inhibition ^d^	In Vitro GST Inhibition ^e^	In Vivo GST Inhibition ^f^
PBO	Reference (MDP)	+++	+++	>30 [11]	+++	+++	−	−
Dillapiol	Parent (MDP)	++	+++	15.8 (Table 1)	++	+++	++	+++
ES1	Ester	+	+	ND	+++	ND	++	ND
ES2	Ester	+	+	ND	+	ND	++	ND
ES3	Ester	++	+++	20.0 (Table 1)	+++	ND	++	ND
ET1	Ether	+	++	ND	+	ND	++	ND
ET2	Ether	+++	+++	27.0 (Table 1)	++	+++	+++	+++
ET3	Ether	++	++	ND	+	ND	+++	++

^a^ Synergism ranking in mortality bioassays based on 24 h mortality at 10 ppm (ingestion) or 50 ppm (topical) pyrethrum with 1:5 synergist ratio: +++: strong (>80% mortality at lowest pyrethrum concentration); ++: moderate (60–80% mortality); +: weak (<60% mortality); ^b^ SR: Synergism Ratio (LC_50_ pyrethrum alone/LC_50_ pyrethrum + synergist). ND: not determined due to limited compound availability; ^c^ In vitro P450 inhibition ranking (Figure 4): +++: activity comparable to PBO (>80% inhibition); ++: moderate inhibition (50–80%); +: weak inhibition (<50%); −: no inhibition; ^d^ In vivo P450 inhibition ranking (Figure 5): +++: significant reduction (*p* < 0.05) compared to untreated RS-CPB; ND: not tested; ^e^ In vitro GST inhibition ranking (Figure 6): +++: strong inhibition (>80%); ++: moderate inhibition (50–80%); –: no inhibition. ET2 IC_50_ = 0.23 mM (180× lower than DEM). ^f^ In vivo GST inhibition ranking (Figure 7): +++: significant reduction (*p* < 0.05) compared to untreated RS-CPB; ++: moderate reduction; –: no reduction; ND: not tested.

## Data Availability

The original contributions presented in this study are included in the article. Further inquiries can be directed to the corresponding author.

## Abbreviations

The following abbreviations are used in this manuscript:

PBO	piperonyl butoxide
CPB	Colorado potato beetle
SS-CPB	insecticide-susceptible CPB strain
RS-CPB	insecticide-resistant CPB strain
MDP	methylenedioxyphenyl
GST	glutathione S-transferase
DEM	diethyl maleate
